# Ethnobotanical Survey of Medicinal Plants Utilized by Traditional Healers in Managing Respiratory Tract Infections in the Kagera Region, Tanzania

**DOI:** 10.1155/tswj/5274014

**Published:** 2026-07-14

**Authors:** Neema Gideon Mogha, Pamela Epaphra Sola, Elbert Anyambilile Mbukwa, Olivia John Kalokora

**Affiliations:** ^1^ Department of Biological Sciences, Dar es Salaam University College of Education (DUCE), University of Dar es Salaam, Dar es Salaam, Tanzania, udsm.ac.tz; ^2^ Department of Chemistry, Dar es Salaam University College of Education (DUCE), University of Dar es Salaam, Dar es Salaam, Tanzania, udsm.ac.tz

**Keywords:** ethnomedicine, medicinal plants, respiratory infections, Tanzania, traditional healers

## Abstract

Respiratory tract infections (RTIs) are the primary cause of morbidity and mortality in many low‐and middle‐income countries. In most of these countries, including Tanzania, medicinal plants (MPs) are primarily used to manage various ailments, though the majority are not yet documented. Therefore, this study is aimed at documenting MPs used by traditional healers (THs) in managing respiratory infections (RTIs) in the Kagera region. A cross‐sectional survey was conducted in the Bukoba, Misenyi and Karagwe districts of the Kagera region using interviews, a semistructured questionnaire and field excursions. Ethnobotanical information on MPs′ local names, plant parts used, preparation methods, route of administration, and threats were documented. Twenty‐eight plant species from 21 families were recorded, whereby Lamiaceae (14.3%) was the dominant family. Out of 28 MPs documented *Ocimum gratissimum* L. (Lamiaceae) had the highest relative frequency of citation (RFC) (0.56), followed by *Eucalyptus camaldulensis* Dehnh. (Myrtaceae) and *Citrus limon* Osbeck (Rutaceae) (0.33 each). Informant consensus factor (ICF) for RTIs was 0.70. Trees (50%) and leaves (57.1%) were the most prevalent growth form and plant part used, respectively. Decoction (64%) was the most common method used to prepare remedies, and most remedies were administered orally (68%). Urbanisation (29%) and agricultural expansion (24%) reported to threaten MPs existence in the study areas. The findings from this study revealed that the local people from Kagera region are using MPs for management of RTIs, and THs have high level of agreements regarding the specific plant species to manage RTIs, which indicated by extensive knowledge of using them. Most of species reported have been proven to possess different bioactive compounds with pharmacological activities. Therefore, plants reported in this study are important candidate for further studies on their safety and toxicology. Moreover, following MP threats reported in this study, education on conservation initiatives is recommended to ensure their survival and sustainable utilisation.

## 1. Introduction

Respiratory tract infections (RTIs) affect the respiratory systems, including nasal passages, bronchi and lungs [1]. In developing countries, RTIs such as asthma, tuberculosis, chronic bronchitis and pneumonia are the major causes of morbidity and mortality [2]. The literature indicates that in 2017, RTIs accounted for approximately 10 million deaths globally [3]. The emergence of the recent pandemic coronavirus disease (COVID‐19) has led to a surge in RTIs such as pneumonia, acute respiratory distress syndrome, septic shock and cardiovascular manifestations [4]. These infections are increasingly becoming significant health problems due to decreased drug efficacy resulting from the rapid development of drug resistance among disease‐causing microorganisms such as bacteria and viruses [5]. Consequently, many people, particularly in developing countries, rely on MPs for the management of infections. In Tanzania, about 60% of the rural population depend on traditional medicine as their core health care support [6]. The high usage of MPs is also associated with their cultural acceptability, less side effects compared with modern drugs and their availability at relatively low or no cost [7].

Following the significant contributions of MPs in the management of RTIs, various documentation studies have been conducted, reporting a diverse number of species. For instance, a study conducted in South Africa reported that 224 plant species have been used in the treatment of RTIs [8]. A review on MPs used against respiratory diseases related to COVID‐19 in African countries recorded a total of 64 species to have been used in Benin Republic, 41 species in Cameroon, 33 species in Morocco and 27 species in Gabon [9]. In Tanzania, several ethnobotanical studies have been conducted to document MPs used for management of RTIs ailments including a study on indigenous knowledge and quantitative analysis of medicinal plants used for treatment of respiratory tract disorders in Mid‐Western Tanzania conducted by [10] from which 42 MPs were reported for treatment of RTIs. An annotated inventory of Tanzanian medicinal plants traditionally used for the treatment of respiratory bacterial infections by [2] documented 169 plant species used by Tanzanian for management of RTIs. A review by Kacholi [11] on antiasthmatic medicinal plants of Tanzania found 62 traditional medicinal plants used for treatment of asthma. Although ethnobotanical studies have been conducted in the country documenting MPs for RTIs, none of them focused solely on documentation of MPs used against RTIs in Kagera region to generate a comprehensive inventory of MPs for managing RTIs. This study is imperative following an increase in respiratory cases associated with COVID‐19, which compromises the immune system [12]. Therefore, the present study was designed to document MPs used for the management of RTIs in Bukoba Urban, Misenyi and Karagwe districts in Kagera region.

## 2. Material and Methods

### 2.1. Study Area

**Figure 1:**
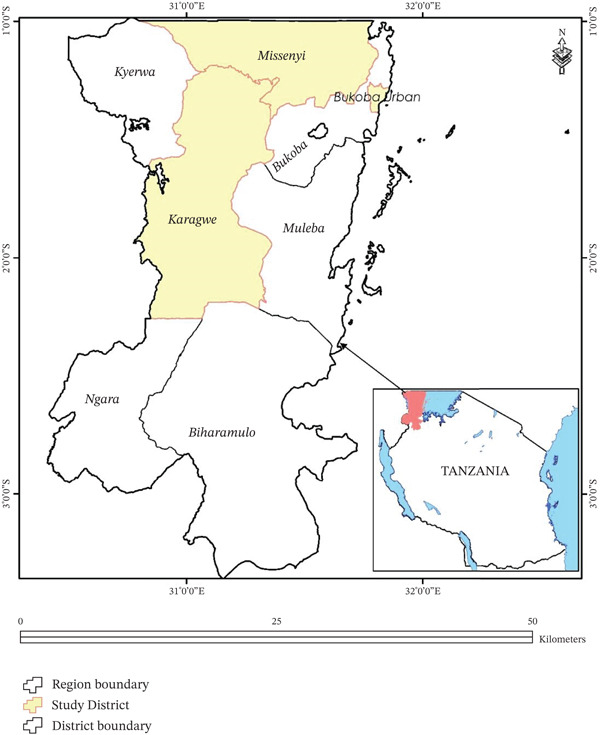
Map showing the three study districts in Kagera region.

The study was conducted in three districts: Bukoba Urban, Misenyi and Karagwe, in the Kagera region (Figure 1). This region lies between latitudes 1°00 ^′^ and 2°45 ^′^ S and longitudes 30°25 ^′^–32°40 ^′^ E in northwestern Tanzania. It is bordered by Rwanda and Burundi to the west, Uganda to the north and Kigoma and Shinyanga regions to the south. To the east, it borders Lake Victoria and Geita region. The region is characterised by a tropical wet and dry savannah climate, featuring a bimodal rainfall pattern in March–May and October–November, with an average rainfall of 245.7 mm and temperatures of 22.04°C [13]. Most residents of Kagera are of Bantu origin, with the Haya tribe being the largest ethnic group, followed by Nyambo, Subi and Hangaza. The main economic activity is the agricultural production of food, such as bananas and beans, alongside commercial crops, including coffee, cotton and tea [13].

### 2.2. The Ethnobotanical Survey

A cross‐sectional ethnobotanical study was conducted from May to June 2021 in three districts of Bukoba urban, Misenyi and Karagwe. A total of 48 THs were identified with the aid of the district medicinal plant coordinator in collaboration with the regional medical officer (RMO). Before data collection commenced, the goal and objectives of the study were communicated to the traditional healers (THs) and their oral consent to participate in this study was obtained. Interviews, semistructured questionnaires and field excursions were used to collect ethnobotanical information on the local names of MPs used for management of RTIs, plant parts used, mode of preparation, route of administration and their threat. Social demographic information of THs (sex, age, education level and their experience in using MPs) was also collected. All MPs mentioned were collected and most of them were identified in the field with the help of an experienced botanist. For few challenging species which could not be immediately identified in the field were pressed for further identification in the herbarium of the Dar es salaam University College of Education. All scientific names were verified using the International Plant Name Index (IPNI) and Plants of the World (POWO) websites.

### 2.3. Data Analysis

Both descriptive and quantitative statistical methods were employed to analyse the respondents′ social demographic information collected. Statistical Package for Social Sciences (SPSS) Version 23 was used to organise and analyse the collected data. The results obtained are presented in figures and tables. The relative frequency of citation (RFC), which is an index to show cultural importance of a species in the area as a medicinal plant, was calculated using the following formula:

RFC=FCN,

whereby FC is the total number of respondents who mentioned the use of plant species for management of RTIs and *N* is the total number of respondents involved in the study.

Informant consensus factor (ICF) index, which is used for testing homogeneity of the informants′ knowledge and acceptance about a certain remedy for particular ailments was calculated using the following formula:

ICF=Nur−NtNur−1,

where ‘Nur’ refers to the total number of use reports for each category of ailment and ‘Nt’ refers to the total number of species used for that category of ailment. Since this study only used one category of disease (RTIs), the ICF was calculated following the given dataN_ur_ = 90andN_t_ = 28.

## 3. Results

### 3.1. TH′s Demographic Profile

A total of 48 THs participated in this study, with 66.7% being male and 33.3% female. Karagwe and Misenyi each had the highest proportion of THs at 37.5%, whereas Bukoba Urban had only 25%. The THs involved in this study varied in age, with most being adults aged between 45 and 64 years (50%), followed by the elderly who were aged 65 years and above (27.1%). The majority of key informants had a primary level of education (72.9%), and most had over 15 years of experience in practising herbalism (35.4%). Details on the demographic characteristics of THs are summarised in Table 1. Statistical analysis results showed no significant difference in MPs knowledge between gender (*t* = 1.517, *p* = 0.07), but the knowledge differed significantly between age groups (*F* = 6.94, *p* = 0.002) whereby THs with 65 years and above were more knowledgeable than others (*p* < 0.05). In terms of education level, the knowledge differed significantly (*F* = 4.035, *p* = 0.01), the illiterate respondents found to have significantly higher MPs knowledge (*p* < 0.05). Regarding the years of experience in herbalism, the significant variation between groups was revealed (*F* = 5.221, *p* = 0.003), whereby those with experience between 11–15 years were found to be more knowledgeable than the others.

**Table 1:** Demographic information of traditional healers.

Districts		Karagwe		Misenyi		Bukoba Urban		Total		Average number of species
**Factors**	**Category**	**n**	**%**	**n**	**%**	**n**	**%**	**n**	**%**	**Average number of species**

Sex	Men	13	72.2	12	66.7	7	58.3	32	66.7	4.78 ± 1.99
	Women	5	27.8	6	33.3	5	41.7	16	33.3	3.62 ± 3.28

Age (years)	25–44	2	11.1	4	22.2	5	41.7	11	22.9	3.18 ± 1.940
	45–64	10	55.6	9	50.0	5	41.7	24	50	3.71 ± 1.76
	≥ 65	6	33.3	5	27.8	2	16.6	13	27.1	6.15 ± 3.00

Education	No education	3	16.7	3	16.7	1	8.3	7	14.6	6.86 ± 2.91
	Primary	15	83.3	15	83.3	5	41.7	35	72.9	5.34 ± 2.68
	Secondary	0	0	0	0	4	33.3	4	8.3	2.25 ± 0.96
	College university	0	0	0	0	2	16.7	2	4.2	1.50 ± 0.71

Experience	< 5 years	1	5.6	3	16.7	2	16.7	6	12.5	2.00 ± 0.89
	5–10 years	1	5.6	4	22.8	5	41.7	10	20.8	5.2 ± 2.35
	11–15 years	6	33.3	5	32	4	33.3	15	31.3	6.27 ± 2.31
	> 15 years	10	55.6	6	33.3	1	8.3	17	35.4	5.4 ± 2.43

### 3.2. Taxonomic Diversity of Medicinal Plants

**Figure 2:**
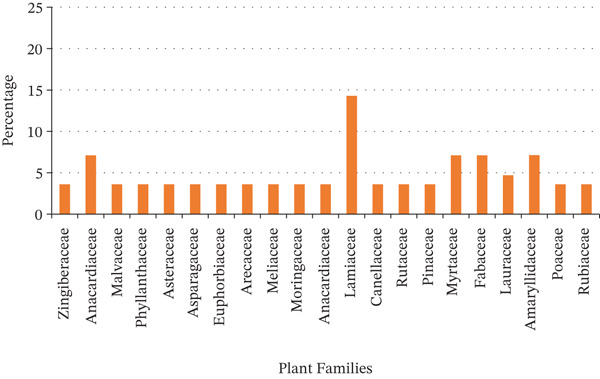
Families of medicinal plants used for management of respiratory tract infections.

Twenty‐eight MPs from 21 families were reported being used by THs in the study areas for management of RTIs. Family Lamiaceae (14.3%) had the highest number of plant species, followed by Anacardiaceae, Myrtaceae, Fabaceae, and Amaryllidaceae (with 7.1% each), whereas the rest of the families had only a small proportion (Figure 2).

Out of 28 medicinal plant species documented in the present study, *Ocimum gratissimum* had the highest RFC (0.56) followed by *Eucalyptus camaldulensis* and *Citrus limon* (0.33, each). The value of the ICF for RTIs, which is the only one category in the collected data for this study, was 0.70. The detailed information on the MPs, their RFC and other associated information is presented in Table 2.

**Table 2:** Ethnobotanical information of medicinal plants used for management of respiratory tract infections.

Voucher number	Scientific name	Local name (haya)	Common name	Family	Growth form	Part used	Ailment cured (haya name)	Preparation mode	Administration route	RFC
KGR 36	*Abrus precatorius* L.	Akaligaligo	Rosary pea	Fabaceae	Climber	Whole plant	Cough (okukolola) and flu (ekiinzi)	Decoction	Oral	0.10
KGR 33	*Allium cepa* L.	Ekitunguru	Onion	Amaryllidaceae	Herb	Bulb	Flu (ekiinzi), cough (okukolola) and COVID‐19 (corona)	Chew	Oral	0.04
KGR 21	*Allium sativum* L.	Ekitunguru swaumu	Garlic	Amaryllidaceae	Herb	Bulb	Flu (Ekiinzi), cough (okukolola) and COVID‐19 (corona)	Chew	Oral	0.06
KGR12	*Azadirachta indica* A.Juss	Omuarobaini	Neem plant	Meliaceae	Tree	Leaves	Asthma (okuniga), COVID‐19 (corona), bronchitis (olufuba), cold (obukonko) and pneumonia (olufuba)	Decoction	Oral/steaming	0.08
KGR04	*Bridelia micrantha* (Hochst.) Baill.	Omushamako	Coastal golden‐leaf	Phyllanthaceae	Tree	Leaves	Cough (okukolola), flu (ekinzii) and sore throat (ebironda bya amalaka)	Decoction	Oral	0.04
KGR10	*Carica papaya* L.	Omupapari	Pawpaw	Caricaceae	Tree	Leaves	Flu (ekinzii), cold (ekinzii)) and COVID‐19 (corona)	Infusion	Steaming	0.08
KGR 27	*Cinnamomum verum* J.Presl.	Omudarasini	Cinnamon	Lauraceae	Tree	Bark	Cough (okukolola)	Decoction	Oral	0.02
KGR 19	*Citrus limon* (L.) Osbeck	Omudimu	Lemon	Rutaceae	Tree	Fruits	Cold (obukonko), cough (okukolola), flu (ekiinzi) and COVID‐19 (corona)	Infusion	Steaming	0.33
KGR 22	*Cupressus lusitanica* Lindl. ex Parl.	Omukrismasi	Mexican cedar	Cupressaceae	Tree	Leaves	Cold (obukonko) and flu (ekiinzi)	Decoction	Steaming	0.02
KGR 06	*Cymbopogon schoenanthus* (L.) Spreng.	Ekijanishamba	Lemon grass	Poaceae	Herb	Leaves	Flu (ekiinzi), cough (okukolola) and COVID‐19 (corona)	Decoction	Oral/steaming	0.31
KGR15	*Dracaena steudneri* Engl.	Omugorogoro	Bush night fighter	Asparagaceae	Shrub	Leaves	Asthma (okuniga), pneumonia (olufuba), chest pain (ebironda bya amalaka) and cough (okukolola)	Infusion	Oral	0.13
KGR 18	*Eriosema psoraloides* Baill.	Omukakara	Canary Pea	Fabaceae	Shrub	Bark	Flu (ekiinzi), chest pain (ebironda bya amalaka) and cough (okukolola)	Decoction	Steaming	0.13
KGR 13	*Eucalyptus camaldulensis* Dehnh.	Omukaritusi	River red gum	Myrtaceae	Tree	Leaves	Clean throat, chest pain (ebironda bya amalaka), flu (ekiinzi), cough (okukolola) and COVID‐19 (corona)	Decoction	Oral	0.33
KGR09	*Gymnanthemum amygdalinum* (Delile) Sch.Bip.	Omubilizi	Congo Bololo	Asteraceae	Shrub	Leaves	Cough (okukolola) and flu (ekiinzi)	Decoction	Oral	0.27
KGR03	*Hibiscus fuscus* Garcke	Omusinga	Hibiscus	Malvaceae	Shrub	Leaves	Asthma (okuniga), cough (okukolola), flu (ekiinzi), bronchitis (olufuba) and cold (obukonko)	Infusion	Oral	0.08
KGR 17	*Mangifera indica* L.	Omunembe	Mango	Anacardiaceae	Tree	Leaves	COVID‐19 (corona), flu (ekiinzi) and cough (okukolola)	Decoction	Steaming	0.10
KGR 30	*Mentha spicata* L.	Omunanaa	Pepper mint	Lamiaceae	Herb	Leaves	COVID‐19 (corona), flu (ekiinzi), bronchitis (olufuba) and asthma (okuniga)	Infusion	Oral	0.04
KGR 26	*Moringa oleifera* Lam.	Omurongo	Drumstick tree	Moringaceae	Tree	Roots	Difficulty in breathing (obutembi), cough, flu (ekiinzi) and COVID‐19 (corona)	Decoction	Oral	0.08
KGR 29	*Ocimum gratissimum* L.	Kashwagara	African basil	Lamiaceae	Shrub	Leaves	Flu (ekiinzi), COVID‐19 (corona), cough (okukolola) and pneumonia (olufuba)	Decoction	Steaming/oral	0.56
KGR 25	*Ocimum suave* W.	Akachumbamwani	Wild basil	Lamiaceae	Shrub	Leaves	Flu (ekiinzi), cough (okukolola) and COVID‐19 (corona)	Infusion	Steaming	0.10
KGR31	*Phoenix reclinata* Jacq.	Omukikimbo	Wild date palm	Arecaceae	Tree	Barks	Flu (ekiinzi), cough (okukolola) and nose congestion (okubanwa)	Decoction	Oral	0.10
KGR08	*Psidium guajava* L.	Omupera	Guava	Myrtaceae	Tree	Leaves	Flu (ekiinzi), bronchitis (olufuba) and cough (okukolola)	Decoction	Oral	0.23
KGR 23	*Rubia cordifolia* L.	Akashabaragesi	Indian madder	Rubiaceae	Climber	Leaves	Asthma (okuniga), cough (okukolola), flu (ekinzii) and bronchitis (olufuba)	Infusion	Oral	0.04
KGR 24	*Shirakiopsis elliptica* (Hochst.) Esser	Omushasha	Milk tree	Euphorbiaceae	Tree	Bark	Cough (okukolola) and flu (ekiinzi)	Decoction	Oral	0.10
KGR02	*Syzygium cumini* (L.) Skeels	Omuzambarau	Java plum	Myrtaceae	Tree	Bark	Cough (okukolola)	Decoction	Oral	0.02
KGR 07	*Tetradenia riparia* (Hochst.) Codd.	Omushunshu	Ginger bush	Lamiaceae	Shrub	Leaves	COVID‐19 (corona), cough (okukolola) and bronchitis(olufuba)	Infusion	Oral	0.27
KGR16	*Warburgia ugandensis* Sprague	Ompilipili	East African greenheart	Canellaceae	Tree	Leaves	Flu (ekiinzi), cough (okukolola) and COVID‐19 (corona)	Decoction	Oral	0.10
KGR01	*Zingiber officinale* Roscoe	Etangawizi	Ginger	Zingiberaceae	Herb	Roots	Cold (obukonko), cough (okukolola), flu (ekiinzi) and COVID‐19 (corona)	Decoction	Oral	0.31

### 3.3. The THs Understanding of the Diseases Under Study

The THs′ understanding and management of RTIs is deeply rooted in indigenous knowledge, experience and cultural belief systems. The THs also known as ‘abafumu’ does not carry out any biomedical diagnosis, but illnesses affecting the respiratory system are usually interpreted through a combination of physical observation, environmental conditions, and spiritual perspectives, reflecting the Haya tribe holistic approach to health and disease. Respiratory ailments are locally recognised by descriptive names that capture their most visible or symptomatic features. The common cold and mild upper respiratory infections are referred to as ‘Obukonko’ or ‘Okufuma omuka’ (literally meaning ‘catching cold air’). Persistent coughing, especially with sputum, is called ‘Okukolola’ (cough), whereas severe cough accompanied by chest pain and difficulty in breathing is termed ‘Okukohola kw′amaafu’ or ‘obutembi bw′amaafu,’ referring to chest or lung‐related illness, often associated with pneumonia or bronchitis. Asthmatic conditions, characterised by wheezing and shortness of breath, are described as ‘Okuniga’ (to suffocate), a condition often believed to result from both environmental exposure to pollutants and hereditary weakness. COVID‐19 is known as corona which is distinguished from all types of RTIs by having combination of symptoms of okuniga and okukohola kw′maafu. In Kagera region like in other areas THs rely on physical examination includes noting the frequency and nature of coughing, sound of breathing, presence of sputum, chest pains, fever and general weakness. The other symptoms involved include colour, odour and texture of sputum, which are carefully evaluated to distinguish between mild infections and severe chest conditions. Traditionally, the management of RTIs is a holistic process that employs multiple strategies, often combining herbal, dietary and ritual components; if the condition persists, more formal medical care may be sought.

### 3.4. RTIs Managed by Medicinal Plants

**Figure 3:**
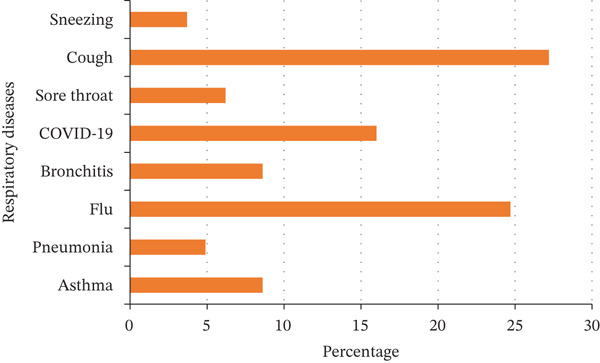
Respiratory tract infections managed using medicinal plants.

Most MPs were utilised for the management of cough (27.2%), flu (24.7%) and COVID‐19 (16.0%) (Figure 3). It was also observed that some were employed to manage five respiratory infections. For instance, *Hibiscus fuscus* Garcke (Malvaceae) was used to managed sore throat, asthma, cough, flu and bronchitis; *Azadirachta indica* A.Juss. (Meliaceae) was used in the treatment of asthma, COVID‐19, bronchitis, sore throat and pneumonia, whereas *E. camaldulensis* Dehnh (Myrtaceae) was utilised to manage sore throat, cough, flu, COVID‐19 and asthma. Others four respiratory infections such as *C. limon* (L.) Osbeck (Rutaceae) was used in the management of cold, cough, flu and COVID‐19, *Mentha spicata* L (Lamiaceae) is used for management of COVID‐19, flu, bronchitis and asthma, whereas *Tetradenia riparia* (Hochst.) Codd (Lamiaceae) was used for management of COVID‐19, cough, bronchitis and flu. The remaining MPs were used to managed three respiratory infections and the least one (Table 2).

### 3.5. Growth Form and Plant Part Used

**Figure 4:**
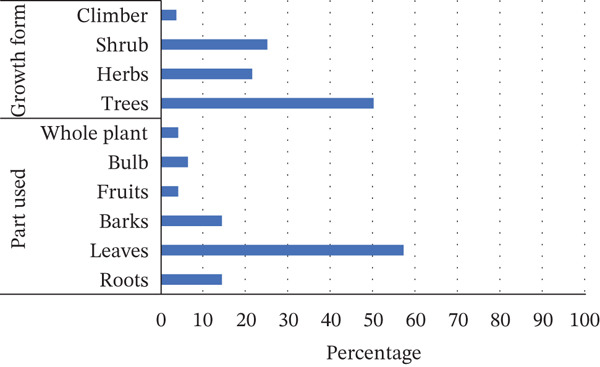
Plant parts used for preparing remedies and their growth forms.

Results from this study revealed that trees were the most dominant growth form (50%), followed by shrubs (25%), whereas the other growth forms had only a few species and contributed a small percentage (Figure 4). In terms of plant parts used to prepare remedies for RTIs, leaves were the most commonly used part (57.1%), followed by roots and barks, each accounting for 14.3%. The other plant parts reported were fruits, bulbs and whole plants, which were rarely mentioned and had relatively low percentage proportions (Figure 4).

### 3.6. Preparation Method and Route of Administration

The findings from this study revealed that THs in the Kagera region use different methods to prepare remedies against RTIs. The most common method reported was decoction (64%), followed by infusion and chewing, which had percentages of 29% and 7%, respectively. It was also found that the oral route was the most prevalent route of administration (68%), followed by steaming inhalation (21%), whereas a small percentage of plants were administered through both oral and steaming (11%).

### 3.7. Similar MPs Managing RTIs in Another Study in the Country

It was found that some of the MPs recorded in this study are used to manage or treat respiratory infections in the other 11 regions of the country. The search results showed that out of 28 MPs documented in this study, 19 MPs (68%) are used in a similar way in other regions of the country. The most dominant MPs are *Zingiber officinale* and *Allium sativum*, which were found to be used in six other regions, followed by *Mangifera indica*, *C. limon* and *Abrus precatorius*, which were found in five regions, and *Allium cepa, Psidium guajava* and *A. indica*, which were found in three to four regions. The results of this similarity of use are indicated in Table 3.

**Table 3:** Reports of Similar MPs used for management of RTIs and their remedies preparation in other regions of Tanzania.

S/N	Family	Species	Part used	Preparation, administration and dosage	Ailment cured	Region	Reference
1	Amaryllidaceae	*Allium cepa* L	Leaves/bulb	Chewing a raw onion twice a day for 3 days. Boil three red onions in 1 L of water, add three tablespoons of honey, and drink twice a day for 2 weeks.	COVID‐19	Dar es Salaam and Morogoro	[6]
			Leaves/bulb	Chop the leaves brand with water to make a juice and use in cooking.	Tuberculosis	Mara	[7]
			Leaves/bulb	ns	COVID‐19	Iringa	[14]
			Leaves/bulb	ns	COVID‐19	Mwanza	[15]
2	Amaryllidaceae	*Allium sativum* L.	Leaves/bulb	Concoction drunk	Asthma	Morogoro	[11]
			Leaves/bulb		COVID‐19	Dar es Salaam and Morogoro	[6]
	Amaryllidaceae	*A. sativum* L.	Leaves/bulb	ns	COVID‐19	Iringa	[14]
			Leaves/bulb	Concoction drunk	Coughs and asthma	Morogoro	[16]
			Leaves/bulb	Concoction drunk	Cough and asthma	Morogoro	[17]
			Leaves/bulb	ns	COVID‐19	Mwanza	[15]
3	Anacardiaceae	*Mangifera indica* L.	Leaves/bark/roots	Decoction drunk	Asthma	Tanga	[11] Mollel
			Leaves	Decoction drunk	Coughs, colds and asthma	Tabora	[10]
			Leaves	Fresh leaves of *M. indica*, *E. globulus* and *T. riparia* boiled in 5 L of water; drink a cup of the mixture and do steam inhalation.	COVID‐19	Dar es Salaam and Morogoro	[6]
			Leaves/bark/roots	Decoction drunk	Asthma and coughs	Morogoro	[17]
			Leaves	ns	COVID‐19	Mwanza	[15]
4	Asparagaceae	*Dracaena steudneri* Engl.	Leaves	Leaves ashes mixed with soda ash and then licked.	Asthma	Kagera Tanzania	[11]
5	Asteraceae	*Gymnanthemum amygdalinum* (Delile) Sch.Bip.	Leaves	ns	Coughs and runny nose	Kagera	[18]
6	Canalleceae	*Warburgia ugandensis* Sprague		ns	Respiratory bacterial infections	Tanzania	[2]
7	Caricaceae	*Carica papaya* L.	Leaves/fruits	Decoction of young leaves drunk for treating COVID‐19. Infusion of flowers is drunk for bronchitis.	COVID‐19 and bronchitis	Tabora	[10]
	Caricaceae	*Carica papaya* L.	Leaves and seeds	Burn leaves and licks, pound seeds and smear.	Coughs, chest and throat pain	Mara	[7]
			Leaves	ns	COVID‐19	Mwanza	[15]
8	Fabaceae	*Abrus precatorius*	Fruit/leaves/roots	Powdered leaf or fruit decoction is mixed with honey or sap and taken orally. Roots are pounded, soaked in coconut juice and then chewed.	Asthma	Kigoma, Kilimanjaro, Tanga and Pwani	[11]
			Roots	Decoction drunk	Coughs	Morogoro	[16]
9	Lamiaceae	*Ocimum gratissimum* L.	Leaves	Fresh leaves chewed for a few minutes and spit out. Fresh leaves boiled in water and drink twice a day.	COVID‐19	Dar es Salaam and Morogoro	[6]
			Leaves	Ns	COVID‐19	Mwanza	[15]
10		*Ocimum suave* W	Leaves	Fresh leaves are boiled in water with *M. oleifera* leaves; drink the decoction and do hot steam inhalation.	COVID‐19	Dar es Salaam and Morogoro	[6]
11		*Tetradenia riparia* (Hochst.) Codd.	Leaves	Fresh leaves of *T. riparia* are chewed for a few minutes and spit out. Chew twice to thrice a day for 14 days.	COVID‐19	Dar es Salaam and Morogoro	[6]
			Leaves	ns	COVID‐19	Mwanza	[15]
12	Lauraceae	*Cinnamomum verum* J.Presl.	Bark	Prepare a herbal tea using six cloves seeds of *S. aromaticum,* bark of *C. verum*, fresh leaves of *M. oleifera* and one spoon of honey in a cup. Drink a cup twice a day for 1 week.	COVID‐19	Dar es Salaam and Morogoro	[6]
13	Meliaceae	*Azadirachta indica* A.Juss	Leaves	Fresh leaves of *A. indica*, *E. globulus* are all boiled in water; drink then inhale it twice a day when sick or once a week for prevention.	COVID‐19	Dar es Salaam and Morogoro	[6]
			Leaves	ns	COVID‐19	Mwanza	[15]
14	Moringaceae	*Moringa oleifera* Lam.	Leaves	ns	Asthma	Morogoro	[11]
				Fresh young leaves of *M. oleifera* boiled in 1 L of water, add two spoons of honey to make herbal tea, and drink a cup twice a day.	COVID‐19	Dar es Salaam and Morogoro	[6]
15	Myrtaceae	*Psidium guajava* L.	Leaves	Infusion drunk	Coughs and influenza	Tabora	[11, 18]
			Leaves	Fresh leaves are boiled in water with *M. oleifera* leaves; drink the decoction and do hot steam inhalation.	COVID‐19	Dar es Salaam and Morogoro	[6]
			Leaves	ns	COVID‐19	Mwanza	[15]
16	Phyllanthaceae	*Bridelia micrantha* (Hochst.) Baill	Roots	Infusion drunk	Coughs	Tabora	[10, 18]
17	Rubiaceae	*Rubia cordifolia* L.	Roots	ns	Respiratory bacterial infection	Tanzania	[2]
18	Rutaceae	*Citrus limon* (L.) Osbeck	Fruits	Decoct, then mix with *A. sativum* then drunk.	Coughs and colds	Tabora	[10, 18]
			Fruits	Make a hot tea of fresh fruits of *C. limon* and *Z. officinale*. Drink while it is warm twice a day.	COVID‐19	Dar es Salaam and Morogoro	[6]
			Leaves/fruits/lemon peel	Mix with ginger, garlic and add hot water then mix with honey, boil in water and boil lemon peel in water then drunk.	Coughs and flue	Mara	[7]
			Fruits	Ns	COVID‐19	Iringa	[14]
			Fruits	Mixed *C.limon* and *Z. officinale* then boil and take hot drink.	COVID‐19	Mwanza	[15]
19	Zingiberaceae	*Zingiber officinale* Roscoe	Whole plant	Decoction drunk	Coughs, influenza, cold, throat infection	Tabora	[10]
			Rhizome/root	Fresh rhizome ground and boiled in water. Add a teaspoon of honey and drink a cup at least three times a day for 7 days. Grind five cloves of *A. sativum* and a piece of *Z. officinale* then add a cup of boiled water and add a tablespoon of honey. Mix them well and drink one tablespoon twice a day for 1 week	COVID‐19	Dar es Salaam and Morogoro	[6]
	Zingiberaceae	*Zingiber officinale* Roscoe	Rhizome/roots	ns	COVID‐19	Iringa	[14]
			Rhizome/roots	Decoction drunk	Cough and asthma	Morogoro	[16]
			Rhizome/roots	Decoction drunk	Coughs	Morogoro	[17]
			Rhizome/roots	Mixed *C. limon* and *Z. officinale* then boil and take hot drink.	COVID‐19	Mwanza	[15]

Phytochemical constituent and pharmacological activities of the studied MPs. Following the importance of the MPs. Literature search was conducted to determine the phytochemical constituents and pharmacological activities of the MPs, which indicate most of the MPs have bioactive compounds that enhance them to be used as MPs as indicated in Table 4.

**Table 4:** Literature on phytochemical constituents and pharmacological activities of some recorded medicinal plants.

Scientific name	Phytochemical compounds	Pharmacological activities	References
*Abrus precatorius* L.	Saponins, phenolics, alkaloids, flavonoids, glycosides, eugenols, steroids, terpenoids, abrin, esters, organic acids and triterpene.	Antihyperlipidaemia, abortifacient, antidiabetic, antiviral, antifertility, anti‐inflammatory, antimicrobial, antioxidant, antiepileptic, antimalarial and antitumor.	[19]
*Allium cepa* L.	Flavonoids includes quercetin and kaempferol, alk(en)yl, cysteine sulfoxides including S‐methyl cysteine sulfoxide and S‐propyl cysteine sulfoxide, cycloalliin, thiosulfinates and sulphides and phenolic compounds.	Antiviral, anticancer, antibacterial, antifungal, lipid modifying, antiobesity, antihypertensive, antidiuretic, antiparasitic, antidiabetic, anti‐inflammatory and antioxidant.	[20, 21]
*Allium sativum* L.	Flavonoid, protocatechic acid, gallic acid, ferulic acid and anthocyanins.	Antiviral, anticancer, antibacterial, antifungal, antidiuretic, antiparasitic, antidiabetic, anti‐inflammatory, antioxidant, hepatoprotective, hypolipidemic, radio and cardio‐protective.	[22]
*Azadirachta indica* A.Juss	Azadirachtin, nimbolinin, nimbin, nimbidin, nimbidol, sodium nimbinate, gedunin, salannin and quercetin. Leaves contain ingredients such as nimbin, nimbanene, 6‐desacetylnimbinene, nimbandiol, nimbolide and ascorbic acid.	Antiviral, antioxidant, antimalaria, anti‐inflammatory, anticancer, immunomodulatory, antihyperglycemic, antiulcers and antibacteria radical scavengers.	[23]
*Bridelia micrantha* (Hochst.) Baill.	Alkaloids, flavonoiis, anthraquinones phenolic compound, triterpenoids, sterols, coumarins, anthocyanins and tannins.	Anti‐inflammatory, antioxidants, antimicrobial, antidiabetic and hepatoprotective effect, anthelmintic, antiamebic, antiviral, antianemic, antibacterial, anticonvulsant, antidiabetic, antidiarrhoeal, antischistosomal, anti‐inflammatory, antiplasmodial, antifungal, antimalarial, antinociceptive and antiviral.	[24, 25]
*Carica papaya* L.	Flavonoids, vitamins, alkaloids, papain, enzymes, carotenoids, steroids, tannins, anthraquinones, cardiac glycosides, phenols, terpenoids, saponins and proanthocynidins.	Anticancer, antimicrobial, antidiabetes, antioxidant, anti‐inflammatory, immunomodulator capacity, improve digestion and wound healing, combatting antibiotic resistant bacteria.	[26, 27]
*Cinnamomum verum* J.Presl.	Cinnamaldehyde, linalool, eugenol, phenols, terpenoids, proanthocyanidins and flavonoids.	Neuroprotective activities, antioxidant, antimicrobial, anti‐inflammatory, antidiabetic, cardiovascular and anticancer.	[28]
*Citrus limon* (L.) Osbeck	Limonoids, flavonoids and phenols.	Antioxidant, anticancer, anti‐inflammatory and antimicrobial.	[28]
*Cupressus lusitanica* Lindl. ex Parl.	Terpenoids (limonene, linalool, 1,8 cineole, sabinene, alpha and beta pinene), polyphenols, camphor, terpenes and umbellulone.	Antimicrobial and antioxidant, cytotoxic activities, antiaging, antifungal, antidermatophytes and anti‐Alzheimer.	[29, 30]
*Cymbopogon schoenanthus* (L.) Spreng.	Elemol, piperitone and 2‐naphthalenemethanol.	Antiplasmodia, antihelmintic, anti‐intestinal, laxative, antiasthma and antipyretic.	[31]
*Dracaena steudneri* Engl	Alkaloids, flavonoids, terpenoids, saponins, tannins, glycosides and phenols.	Anti‐inflammatory, antiallergenic, antiviral, antioxidant, anticarcinogenic, cardiotonic, antidiabetic, antifungal, antiplasmodial, oxytocic, antiprotozoal and antimicrobial.	[32]
*Eucalyptus camaldulensis* Dehnh	Essential oils (p‐cymene, aromadendrene), cineole compounds, monoterpene and sesquiterpene.	Relieve cough, breathing disorders including COVID‐19 and SARS. Anti‐*Candida albicans*, antimalaria, asthma, antimicrobial and antioxidant.	[14]
*Gymnanthemum amygdalinum* (Delile) Sch.Bip.	Flavonoids, alkaloids, terpenes, saponins, tannins, triterpenoids, glycosides steroidal, phenolics and many sesquiterpenes lactones.	Antifungal, antibacteria, cathartic, antifertility, antioxidant, antiallergic, laxative, antithrombotic, hypoglycemic, antimalaria, anticancer, anti‐inflammatory and antidiabetes.	[33–35]
*Hibiscus fuscus* Garcke		Antimicrobial	[36]
*Mangifera indica* L.	Mangiferin, phenolic acid, benzophenones, flavonois, ascorbic acid, carotenoids and tocopherols.	Anticancer, antioxidant, antimicrobial, antidiarrheal and hepatoprotective.	[37]
*Mentha spicata* L.	Flavonoids, carvone and phenolic acids.	Antioxidants, anti‐inflammable and antimicrobial.	[38]
*Moringa oleifera* Lam.	Flavonoids, anthocyanins, steroids, tannic acid, alkaloids, isothiocyanates, saponins, terpenoids, anthraquinone, cardiac glycosides and essential oils.	Hepatoprotective, antihypertensive, cholesterol‐lowering, antiurolithiasis, antifertility, antidiabetic, and antioxidant, nutraceutical and antimicrobial.	[39]
*Ocimum gratissimum* L.	Essential oils, oleanolic acid, caffeic acid, eugenol, thymol, ellagic acid, epicatechin, sinapic acid, rosmarinic acid, chlorogenic acid, luteolin, apigenin, nepetoidin, xanthomicrol, nevadensin, salvigenin, gallic acid, catechin, quercetin, rutin and kaempferol.	Antioxidant, anti‐inflammatory, anticancer, hepatoprotective, antidiabetic, antidiarrheal and antimicrobial.	[40]
*Ocimum suave* W.	Tannins, flavonoids, saponins, steroids alkaloids and phenolics.	Antimicrobial	[41]
*Phoenix reclinata* Jacq.	Phenolic compound, alkaloids, flavonoids, tannins, sterols, saponins.	Nephroprotective, antimalaria, antipyretic, antioxidant, anti‐inflammatory, terpernoids and glycosides.	[42]
*Psidium guajava* L.	Triterpenoids, alkaloids, steroids, tannins, glycosides, flavonoids, and saponins.	Antidiabetic, antidiarrheal, hepatoprotective, anticancer, antioxidant, anti‐inflammatory, antimicrobial, antiallergy and antiplasmodial effects.	[43]
*Rubia cordifolia* L.	Glycosides, resins, alkaloids, oleoresins, quionones, sesquiterpene, lactones saponins and oils (essential and fixed).	Anti‐inflammatory, radioprotectivity, antitumour activities, hepatoprotective activities, antiallergic activities, antiproliferative activities and antiplatelets activities, antimicrobial, antioxidant, antidiabetic and anticonvulsant activity.	[44]
*Syzygium cumini* (L.) Skeels	Polyphenols, flavonoids, anthocyanins, steroids, fatty oils, sterculic acid and linoleic acid.	Antioxidant, iron, vitamin C, antihypertensive, antihyperglycemic effect, antiulcerative, antilipidemic, antisterility, antimicrobial, anti‐inflammatory and antidiabetic, antianemia, gastroprotective, CNS stimulator, antioxidant, anticancer, menoharrgia and cardioprotective and hypolipidemic.	[45]
*Tetradenia riparia* (Hochst.) Codd.	Flavonoid, campesterol, phenols, tannins, stigmasterol and sitosterol.	Anti‐inflammatory, antifungal, larvicidal, antioxidant, antitumor, anthelmintic, antispasmodic, anthelmintic and antimicrobial, antibacterial, insecticidal and antiparasitic.	[46]
*Warburgia ugandensis* Sprague	Sesquiterpenes, flavonoid, polygodial caseamemin lignanamides, muzigadial, warburganal, macrocyclic, cinnamolide‐3*β*‐acetate glycoside, albicanyl acetate and ugandensidial.	Antifeedant, antiulcers, antimycobacterial, antifungal, anti‐inflammatory, antiplasmodial, antiasthmatic, antihelmintic, antileshmanial, antioxidant, antitumor and cytotoxicity.	[47]
*Zingiber officinale* Roscoe	Zingerene tannins, flavonoids, steroids, presence if essential oil and shogaolsm, zingerone, and alkaloids, phenolic compounds and terpenes, gingerols commonly 6‐ gingerols, 8‐ gingerols and 10‐ gingerols.	Neuroprotective, antibacterial, antidiabetic, antimicrobial, antinausea, antioxidants, anti‐inflammatory and pain relieving (antinociceptive) and anticancer.	[48]

### 3.8. Threats to Medicinal Plants

**Figure 5:**
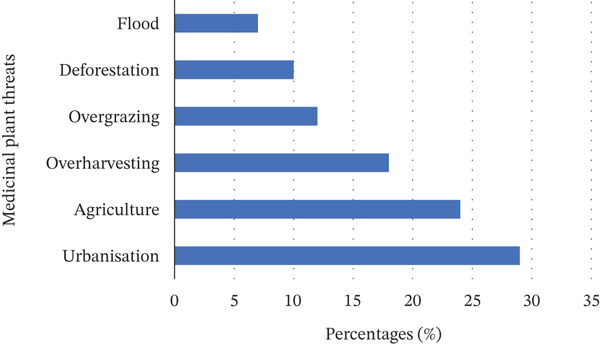
Threats to medicinal plants.

The THs in the Kagera region identified six major factors threatening MPs. The primary threats to MPs highlighted by most THs were urbanisation (29%), expansion of agricultural areas (24%) and overharvesting (18%). Other threats mentioned included overgrazing, deforestation and floods, which were noted by a smaller percentage of THs (Figure 5). During this study, the THs stressed that, given the limited availability of MPs currently experienced, conservation initiatives through domestication by planting MPs in home gardens, on banana farms and in sacks are essential.

## 4. Discussion

### 4.1. Social Demographic Data

The majority of the THs in this study were men. The prevalence of men practising herbal remedies may be linked to the African belief that THs should primarily be male, as they are often regarded as the heads of the family. A similar finding was reported in the Tabora region of Tanzania [49] and Uganda [50]. Most participants were elderly, over 45 years old, which correlates with increased knowledge of MPs alongside years of experience [7]. The prevalence of THs with iliterace or no formal education is likely because this group spends much of their time in local environments with elders who typically utilise MPs, thereby acquiring more knowledge about them. The higher number of elderly individuals compared to younger ones may also be attributed to young people′s preference for modern medicine as they migrate to urban areas for education and employment [49]. Surprisingly, THs with experience of 11–15 had more knowledge compared to those with experience above 15 years this can be attributed to that fact. The young generation have access to formal education and access to wider academic knowledge through schools or college education and also by being able to search information on the media or learning from other areas. The other reason for THs with experience of 11–15 years being more knowledgeable could be attributed by their abilities to communicate with other people from other regions or districts which broaden their understanding of medicinal plants for management various diseases, which is difficulty for old people who only share the knowledge about medicinal plants orally and to the loved one. The other reason could be sharing information through the unions of THs, since in Kagera region currently there are associations of THs in each district and these have an opportunity of meeting and discussing various MPs used for management diseases, which in the past where not existing.

Most MPs used by THs to prepare remedies for RTIs were from the Lamiaceae family. The high use of the Lamiaceae family in this study may be attributed to the presence of various essential oils, which exhibit antiviral activity against SARS‐CoV‐2 and other respiratory tract disorders [51]. Members of the Lamiaceae family possess pharmacological effects, including antioxidant [52, 53], antibacterial, [54] antimalarial [55, 56] and antifungal properties [52, 57]. The prevalence of Lamiaceae in the treatment of RTIs has also been reported in previous studies [56, 58].

The results showed *O. gratissimum* to have the highest value of RFC compared to other MPs collected in this study. The value of RFC, which is 0.56, indicated that almost half of the THs are aware of this species and acknowledge its users for managing RTIs. This is also justifiable in the field where the species was easily accessible and found in most places in the studied sites; hence, it was frequently used. The results of RFC showed the lowest value for *H. fuscus* and *A. indica*, both having 0.08, which are MPs managing more than four RTIs but with the lowest RFC. *A. indica* is the exotic plant species, which is not yet much established in Kagera region; hence, it is only found in a limited place and only known by a few THs as a medicinal plant of important value. *H. fuscus* is a native species in the country but dominated in Afro‐montane and montane areas, which is different from the studied region that has a tropical equatorial climate; therefore, it is also limited and not easily accessible and recognised by many THs, which could be the reason why the species is less abundant and there is little information about this species in the three districts studied in Kagera region. These results are comparable to the results of an ethnobotanical study of wild plants in Northwest Morocco, where some useful plants had the lowest RFC [59] However, this could not be a reason why the species of *H. fuscus* should not proceed for further analysis in the laboratory because it is regarded as managing more than four RTIs, as indicated by a few THs who know about it.

The value of ICF for RTIs was 0.70 which indicates a moderately high level of agreement among local THs regarding specific species used to manage RTIs. It shows the presence of rich traditional knowledge in the community. This strong shared traditional knowledge base within the local community implies that THs have a culture which has relatively well‐defined criteria for treating RTIs. Since the index value obtained indicates clear community agreement on the use of MPs for management of RTIs, therefore the MPs, which have the ability to manage more than three RTIs, have a higher chance of containing active, effective phytochemical compounds which can be identified in the laboratory for future manufacturing of the drugs. The results of ICF were similar to the studies in Cameroon and Ethiopia [60, 61] in which both of them obtained higher values in MPs for treating RTIs.

Three plant species, namely *H. fuscus*, *A. indica*, and *E. camaldulensis* were utilised to manage more than four RTIs which could be attributed by possession of diverse bioactive components with various pharmacological effects against different disease‐causing agents. For instance, *E. camaldulensis* contains essential oils with antiviral, antimicrobial, and antioxidant properties [62, 63]. The essential oil from *Eucalyptus* has been reported to be used in Tunisian folk medicine for treating various RTIs, including bronchitis, pharyngitis, and sinusitis [64]. *A. indica* leaves contain various phytochemical compounds such as flavonoids, alkaloids, phenolic compounds and terpenoids reported to have therapeutic potential against infections [65]. *A. indica* also possess essential constituents such as azadirachtin, nimbolinin, 6‐desacetylnimbinene, ascorbic acid, and 17‐hydroxyazadiradione reported to have therapeutic implications for the prevention and management of infections [66]. More information on bioactive compounds and pharmacological activities of MPs reported in this study presented in Table 3.

Some MPs documented in the present study were report in other studies conducted in Tanzania to have been used for treatment of various respiratory disorders. A study conducted in Morogoro region by [16] showed the species of *A. sativum*, *O. gratissimum* and *Z. officinale* were used against cough and asthma., A study conducted by Mlozi in Iringa region found *A. cepa*, *A. sativum* and *Z. officinale* for treatment of COVID‐19 [14]. The study by [17] in Kimboza forest located in Morogoro Region reported found the species of *A. sativum*, *M. indica* and *Z. officinale* to be used in treatment of RTIs. Several studies reported diverse number of MPs to have been used in management of COVID‐19, for instance; a study conducted in Mwanza by [15] reported the use of *A. cepa*, *A. sativum*, *A. indica*, *C. limon*, *M. indica*, *O. gratissimum*, *P. guajava*, *T. riparia* and Z. *officinale*. Another study conducted in Morogoro Tanzania by [6] reported the species of *A. cepa*, *A. sativum*, *A. indica*, *C. limon*, *M. indica*, *O. gratissimum*, *P. guajava*, *T. riparia* and *Z. officinale* were used in management of COVID‐19, and the study by Charwi in Mara Region [7] documented the use of *A. cepa*, *C. limon* and *C. papaya for treatment of tuberculosis,* coughing, chest throat and flu The species of *A. sativum*, *M. indica*, *Z. officinale*, *C. limon, A. cepa*, *P. guajava* and others, which were, frequently, reporting to be used against RTIs suggests possession of bioactive compounds with therapeutic potential against pathogens affecting respiratory system. Therefore, these plants are consistently relied upon by THs, suggesting perceived effectiveness and cultural trust. Therefore, these plants can be subjected to phytochemical screening, bioassays, and pharmacological evaluations to identify rich in bioactive compounds with antimicrobial, anti‐inflammatory or immunomodulatory properties. Ultimately, such systematic knowledge accelerates the selection of promising species for further scientific validation, guiding the development of safe, effective, and culturally acceptable plant‐based drugs against RTIs.

Trees were the most dominant growth form, and leaves were the preferred plant parts reported for use in remedy preparation. The predominance of trees can be explained by the fact that seasonal variations do not influence them; therefore, they can be accessed throughout the year for medicinal purposes, as previously pointed out by [54, 64]. Compared with other plant parts, the high preference for leaves in the preparation of herbal remedies could be attributed to their abundance of bioactive ingredients effective against diseases [67]. Leaves can be easily harvested in large quantities within a short period. Furthermore, leaves are readily accessible and have a high renewable potential, meaning their high harvesting rate does not lead to MPs′ extinction [56]. Thus, harvesting leaves should be encouraged among local communities as a means of conserving MPs. The prevalent use of leaves compared with other plant parts has also been reported in other studies [1, 68].

Decoction and oral administration were the most preferred methods of remedy preparation and routes of administration, respectively. The high prevalence of decoction is due to its ability to enhance the efficiency of bioactive component extraction from plant materials, detoxify harmful compounds, and sterilise the extracts [69]. Decoction has also been reported as the most common method used to prepare remedies for respiratory diseases in other countries, such as Kenya [23] and South Africa [1]. The widespread use of the oral route can be attributed to the fact that RTIs occur internally, and therefore, oral intake increases the likelihood of reaching the targeted pathogens for curative purposes. These results are consistent with the previous study by [64].

Agricultural expansion and overharvesting were the primary threats to MPs reported by THs. The prevalence of agricultural expansion can be attributed to the fact that most residents are farmers who tend to clear bushes to create more space for cultivation, a situation that was also noted during fieldwork. Agricultural expansion was likewise a significant threat to MPs conservation initiatives in Ethiopia [70]. Conversely, overharvesting can be linked to the Kagera region′s rich diversity of MPs and the residents′ extensive knowledge of these plants and their uses. This knowledge arises from their long‐term interactions and intermarriages with other tribes from neighbouring countries such as Uganda, Burundi and Rwanda [71]. The study further asserted that the extensive harvesting of MPs for commercial purposes has led to the unavailability of certain species, thereby imposing a burden on Indigenous people who have relied on them for medicinal purposes for millennia.

Threats reported in this study align with another study conducted in Tanzania by [49]. Overharvesting of MPs may be linked to the increased demand for MPs due to cultural beliefs, lack of financial capacity to afford modern treatments and scarcity of healthcare services. Massive harvesting of MPs could potentially lead to the disappearance of highly utilised species, thereby highlighting the need for conservation initiatives. The THs further indicated that they have begun conserving MPs through domestication by planting them in their banana farms and surrounding home gardens. Promoting domestication as an essential indigenous method for preserving plant biodiversity was noted in a previous study conducted in Usambara, Tanzania, by [72]. To enhance the conservation of MPs, this study recommends that all stakeholders in MPs should collaboratively participate in educating the public on the importance of sustainable use and protection of MPs for future generations.

## 5. Conclusion

The findings from this study revealed that the Kagera region is abundant in MPs and THs possess extensive knowledge of using them to manage RTIs. THs have strong shared traditional knowledge which implies that the local healing culture has relatively well‐defined criteria for treating RTIs. Many species have been proven to possess different bioactive compounds with pharmacological activities. Therefore, plants reported in this study are important candidates for further studies on their safety and toxicology. Moreover, following MP threats reported in this study, education on conservation initiatives is recommended to ensure their survival and sustainable utilisation.

## Author Contributions

N.G.M. and O.J.K. conceptualised and designed the study. N.G.M., P.E.S., O.J.K. and E.A.M. conducted data collection, analysis and interpretation. N.G.M. drafted the initial manuscript.

## Funding

This study was supported by the University of Dar es Salaam College of Education (DUCE‐21015).

## Disclosure

O.J.K., P.E.S. and E.A.M. read, reviewed, and approved the submission of the final version of the manuscript for publication consideration.

## Ethics Statement

Before starting data collection, the traditional healers were discussed to introduce the project and seek their voluntary participation. Voluntarily, verbal consent from all 48 traditional healers (THs) was obtained after being assured that the information gathered in this study would be used for academic purposes. Before the study commenced, research Ethics for this study (Reference Number AB 3/12(B)) were obtained from the office of the Vice Chancellor of the University of Dar es Salaam.

## Conflicts of Interest

The authors declare no conflicts of interest.

## Data Availability

This article includes all data collected and analysed during this study, presented in tables and figures. Plant specimens collected from the field were pressed and deposited in the herbarium at Dar es Salaam University College of Education, Biological Science Department.
